# Metabolic dysfunction in Friedreich ataxia

**DOI:** 10.1098/rsob.260221

**Published:** 2026-11-11

**Authors:** David Lynch, Clementina Mesaros, Elizabeth Mercado Ayon, Yi Na Dong

**Affiliations:** ^1^ Department of Neurology, University of Pennsylvania, Philadelphia, PA, USA; ^2^ Department of Pharmacology, School of Medicine, University of Pennsylvania, Philadelphia, PA, USA; ^3^ Department of Neurology, School of Medicine, University of Pennsylvania, Philadelphia, PA, USA; ^4^ School of Medicine, University of Pennsylvania, Philadelphia, PA, USA

**Keywords:** lipid, carbohydrate, ceramide, iron–sulfur cluster

## Abstract

Friedreich ataxia (FRDA) is a metabolic disease with two faces. Loss of iron–sulfur cluster biogenesis disables oxidative phosphorylation, the Krebs cycle and the oxidation of fatty acids and ketone bodies, forcing tissues onto glycolysis and stranding lipid in droplets and as ceramide. The second aspect is on the supply side. A frataxin-deficient liver and adipose tissue deliver inadequate lipoprotein, fatty acid and ketone cargo to the heart, skeletal muscle and nervous system, the organs that depend most on imported fuel. Together, these lesions produce a cardiomyopathy that behaves like an engine out of fuel, a mixed diabetes of insulin deficiency and insulin resistance, low lean and bone mass and a redox-collapsed, ferroptosis-prone state. This review traces that logic from the cell to the organ to the whole body. The same map defines a compact panel of blood-based biomarkers, anchored on frataxin protein itself and extended by chain-resolved ceramides and by the succinate : fumarate, BHB : acetoacetate, NAD+ : NADH and GSH : GSSG ratios. It also nominates targets that do not require restoration of frataxin, including NRF2 activation, ferroptosis inhibition, NAD+ and sirtuin repletion, PPARgamma agonism, anaplerosis and ketone-based therapy. Reframing FRDA as a disorder of fuel logistics, rather than of fuel combustion alone, explains why a globally reduced protein produces a selective yet multisystem disease.

## 1. Introduction

Friedreich ataxia (FRDA) is an autosomal recessive neurodegenerative disorder associated with ataxia, gait disorder, areflexia, sensory deficits, optic neuropathy, auditory dysfunction and metabolic myopathy [1–5]. In addition, fatigue itself is a devastating feature of the disorder [6–8]. Outside the nervous system, patients develop cardiomyopathy, scoliosis and variably diabetes. Mutations in the frataxin (*FXN*) gene, predominantly GAA triplet-repeat expansions in intron 1 (96%), cause FRDA, with all pathogenic mutations leading to decreased levels of functional frataxin [1]. GAA repeats suppress transcription of *FXN* mRNA in a manner dependent on the length of the GAA repeat [9,10]. About 4% of FRDA patients carry a GAA expansion in combination with deletion or missense mutations; these typically are null mutations, although some mutations could lead to partially functional proteins [11–13].

*FXN*, a mitochondrially targeted protein, facilitates the synthesis of iron–sulfur clusters, which are used as prosthetic groups in many cellular enzymes [14,15]. Although traditionally associated with the electron transport chain (ETC) and mitochondrial enzymes of the TCA cycle, Fe–S clusters are also used in many other cellular enzymes, including those involved in DNA repair, transport processes and heme synthesis [14,15]. Thus, without normal levels of *FXN*, many bioenergetic processes may be impaired. Classical approaches to the pathophysiology of FRDA have focused on mitochondrial components including iron accumulation in mitochondria, increased oxidative stress and ATP deficiency [16–19]. Less appreciated issues associated with Fe–S cluster deficiency include abnormalities of DNA repair, cytosolic iron deficiency and a large number of downstream metabolic events [14,15]. While ATP deficiency is readily demonstrable in FRDA patients, reactive oxygen species (ROS) production is not as well demonstrated *in vivo* as *in vitro* [20–22]. Thus, the pathological events of FRDA may reflect some of the chronic events downstream from Fe–S deficiency, consequent ATP deficiency and other metabolic changes.

The tissue distribution of FRDA aids in understanding the pathogenesis of the disease. FRDA impacts highly active metabolic tissues such as the heart and the brain that also use non-glucose related fuels (fatty acids and ketones). Frataxin-induced abnormalities in the metabolism of fatty acids and ketones thus might contribute to the pathophysiology, particularly in conjunction with the failure of ATP production noted in FRDA. Consistent with failure of energy production, patients with FRDA are pathologically small in many situations, like patients with mitochondrial disease and global metabolic dysfunction [23–26]. This reflects not only a decrease in muscle size but also increased fat and decreased bone density [23,24]. The anthropometric profile of FRDA suggests that it reflects a global disease as much as a tissue specific disease.

Whole-body metabolism in FRDA bears the imprint of the same energy deficit (figure 1). In frataxin-deficient skeletal muscle, this is accompanied by activation of the integrated stress response and loss of lean mass, indicating that muscle is not merely underused but actively remodelled by impaired oxidative metabolism [23,27]. Because skeletal muscle is a major site of fatty acid and glucose disposal, its metabolic remodelling feeds back on systemic insulin sensitivity and substrate availability, linking the cellular lesion to the anthropometric phenotype of the disease. In this review, we highlight the metabolic features of FRDA at the cellular, organ and systems level to propose ideas on how such features contribute to the dysfunction in FRDA.

**Figure 1.**
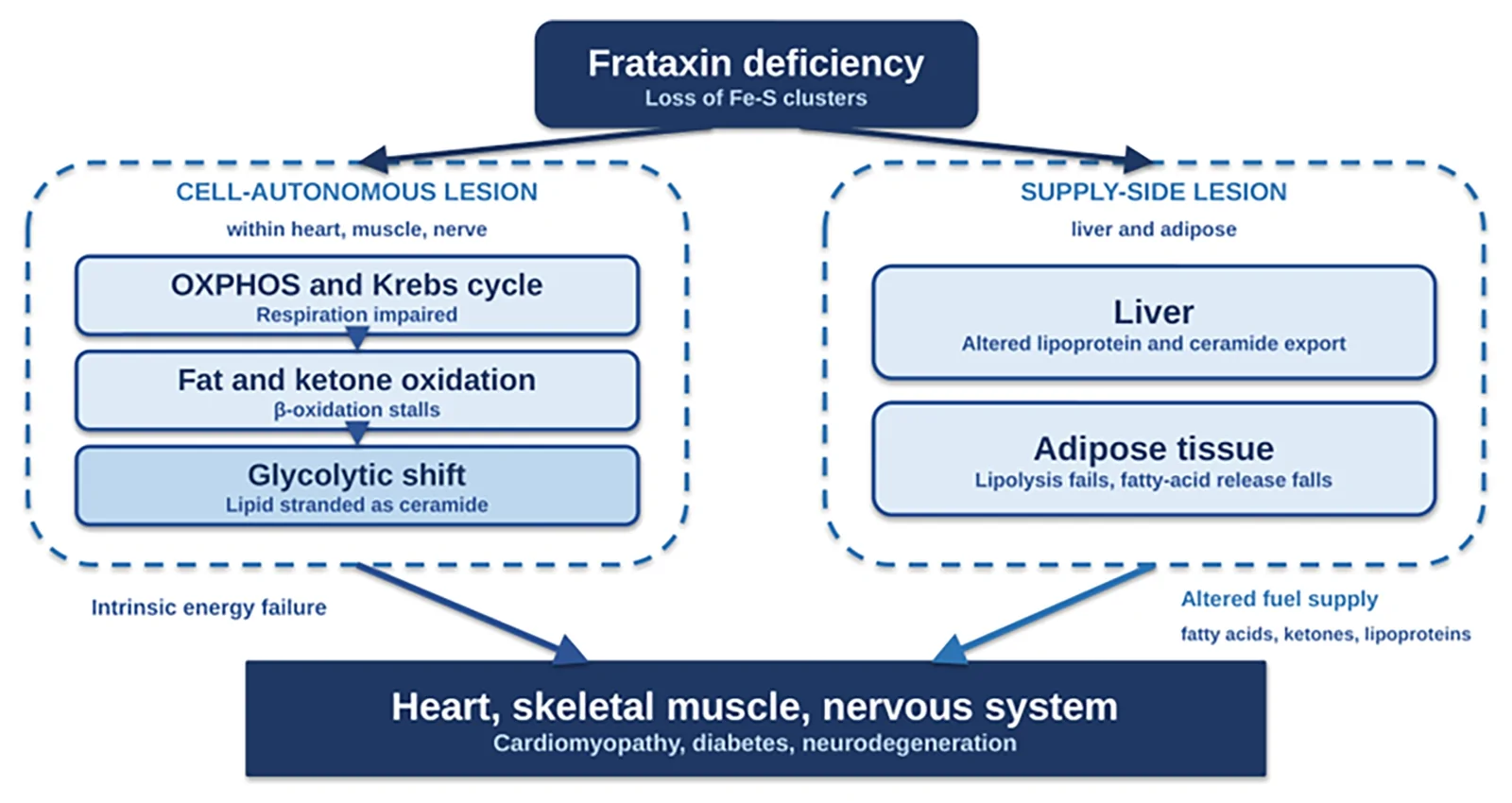
The two-level model of metabolic dysfunction in Friedreich ataxia, a cell-autonomous lesion within the heart, skeletal muscle and nervous system together with a supply-side lesion in the liver and adipose tissue. Fe–S, iron–sulfur; OXPHOS, oxidative phosphorylation.

## 2. Historical aspects

Nikolaus Friedreich described the disorder in a series of reports between 1863 and 1877, and the causal GAA triplet-repeat expansion in *FXN* was identified in 1996 [28]. FRDA is the most common inherited ataxia, with an average global prevalence of about 0.5 cases per 100 000 people [1]. Prevalence is highest in populations of European and Indian ancestry, approaching one case per 30 000 people in Australia, India and parts of Europe. It falls to one to three cases per 100 000 people in North America and to below one case per 100 000 in Mexico, Central America and South America, and the disease is very rare in sub-Saharan Africa, People's Republic of China, Japan and southeast Asia [1]. Within Europe there is a marked gradient, from one case per 20 000 people in the southwest to one case per 250 000 in the north and east [1]. Onset is usually before age 25 years and most often between 8 and 15 years, longer GAA1 repeats predict earlier onset, mean life expectancy is 35–40 years and cardiac disease is the leading cause of death [1]. These figures set the scale of the clinical problem. They also explain why the pregenetic literature described below rested on small and often diagnostically heterogeneous cohorts, and why its inconsistencies are unsurprising in retrospect.

Before the discovery of the exact mutation in FRDA, many different forms of metabolic dysfunction had been identified in the disease, though such findings were reproduced inconsistently and may have come from individuals with incorrect diagnoses. The earliest studies [29] identified loss of glycolytic enzymes in relevant cells: nucleus dorsalis of Clarke, deep cerebellar nuclei and Purkinje cells. Similarly, in some studies, pyruvate oxidation was decreased and seemingly confirmed by pyruvate loading tests [30]. Such abnormalities were associated with the lipoamide dehydrogenase component of pyruvate dehydrogenase [31,32]. A hyperbilirubinemia similar to Gilbert syndrome appeared to occur, a deficiency in protoporphyrin appeared in some studies, and selected amino acids were altered in the cerebrospinal fluid (CSF) or plasma, mainly alanine, aspartate and taurine [33–35]. However, other studies (largely from the large cohort in Quebec) did not confirm such results [36–38].

A second area of pregenetic investigation was plasma lipid and lipoprotein levels. Although no difference was observed in cholesterol levels, high density lipoprotein (HDL) levels decreased compared to controls [39,40]. Other studies suggested low density lipoprotein (LDL) abnormalities and increases in saturated fats [41]. Such findings were associated with abnormal features of erythrocyte membranes including altered phospholipid composition, and lipoprotein lipase levels appeared deficient in vessels from FRDA tissue [42]. However, such abnormalities were inconsistently reproduced across different cohorts and under different conditions. Interestingly, many (but not all) of the findings on FRDA before the advent of genetic testing have been reproduced in recent studies, but others have not been readdressed. The following discussion will consider where such early findings contribute to the pathophysiology of FRDA and their relationship with the normal function of frataxin.

## 3. Cellular autonomous metabolism in Friedreich ataxia

### 3.1. Carbohydrates, the Krebs cycle and energy failure

As the major function of frataxin is facilitating the synthesis of Fe–S clusters, the events in FRDA mirror the loss of the most well-known iron–sulfur cluster enzymes, those of the electron transport chain and Krebs cycle. Complexes I, II and III all use Fe–S clusters, and these enzymes are structurally associated with frataxin [43–48]. Thus, its deficiency logically leads to functional deficits in ATP production. Over the long term, levels of these enzymes are likely to be downregulated, and imaging studies suggest that complex I levels are low in the brain in FRDA patients and in FRDA mouse models [49,50]. In addition, mitochondrial dysfunction from abnormalities of electron transport should lead to production of ROS [51]. As transport of electrons through the ETC provides the final steps in oxidative metabolism of all substrates, there will be metabolic effects on the disposition of carbohydrates, fats and proteins in the cell.

Similar diffuse metabolic effects will result from TCA cycle dysfunction in FRDA. Aconitase and succinate dehydrogenase both use Fe–S clusters, and their activity levels decrease in FRDA [43–50,52]. This should lead to a stalling of the cycle at certain points with increases in metabolites proximal to the block [53]. In addition, frataxin deficiency gives rise to a secondary decrease in the enzyme OXCT1 in cells [54]. This enzyme controls entry of ketone bodies into the TCA cycle; thus, FRDA patients should have difficulty with ketolysis. Clinically, this may explain the relative absence of proprioceptive neurons in OXCT1 knock-out mice, as they selectively require ketolysis for development [55].

The reach of Fe–S cluster loss extends well beyond the canonical respiratory and Krebs-cycle enzymes. Reduced pyruvate oxidation and diminished entry of glucose-derived carbon into the Krebs cycle force frataxin-deficient cells to rely disproportionately on glycolysis for ATP, a shift accompanied by elevated lactate and by the downregulation of the mitochondrial solute carriers that would otherwise sustain anaplerotic flux [56]. Fatty acid oxidation is similarly constrained, because several of its enzymes are themselves Fe–S dependent or rely on Fe–S-derived co-factors, including electron-transfer-flavoprotein-ubiquinone oxidoreductase and the lipoate and biotin synthases that supply prosthetic groups to pyruvate dehydrogenase and the carboxylases. Consistent with a stalled but persistently engaged β-oxidation pathway, FRDA patient fibroblasts accumulate HMG-CoA, acyl-carnitines and β-hydroxybutyryl-CoA, biochemical signatures of fatty acids entering oxidation faster than their products can be cleared [57]. The net effect is a loss of metabolic flexibility: tissues that normally toggle among glucose, fatty acids and ketones become progressively confined to the least efficient of these fuels. The iron–sulfur-dependent enzymes most affected by frataxin deficiency span every fuel pathway, the electron transport chain complexes I–III, aconitase and succinate dehydrogenase in the Krebs cycle, the lipoate and biotin synthases that supply pyruvate dehydrogenase and the carboxylases, electron-transfer-flavoprotein-ubiquinone oxidoreductase in β-oxidation and OXCT1 in ketolysis. Their shared loss drives the glycolytic shift, the stalling of β-oxidation with lipid-droplet accumulation, and the downstream ATP deficit, oxidative stress and ferroptosis susceptibility (figure 2).

**Figure 2.**
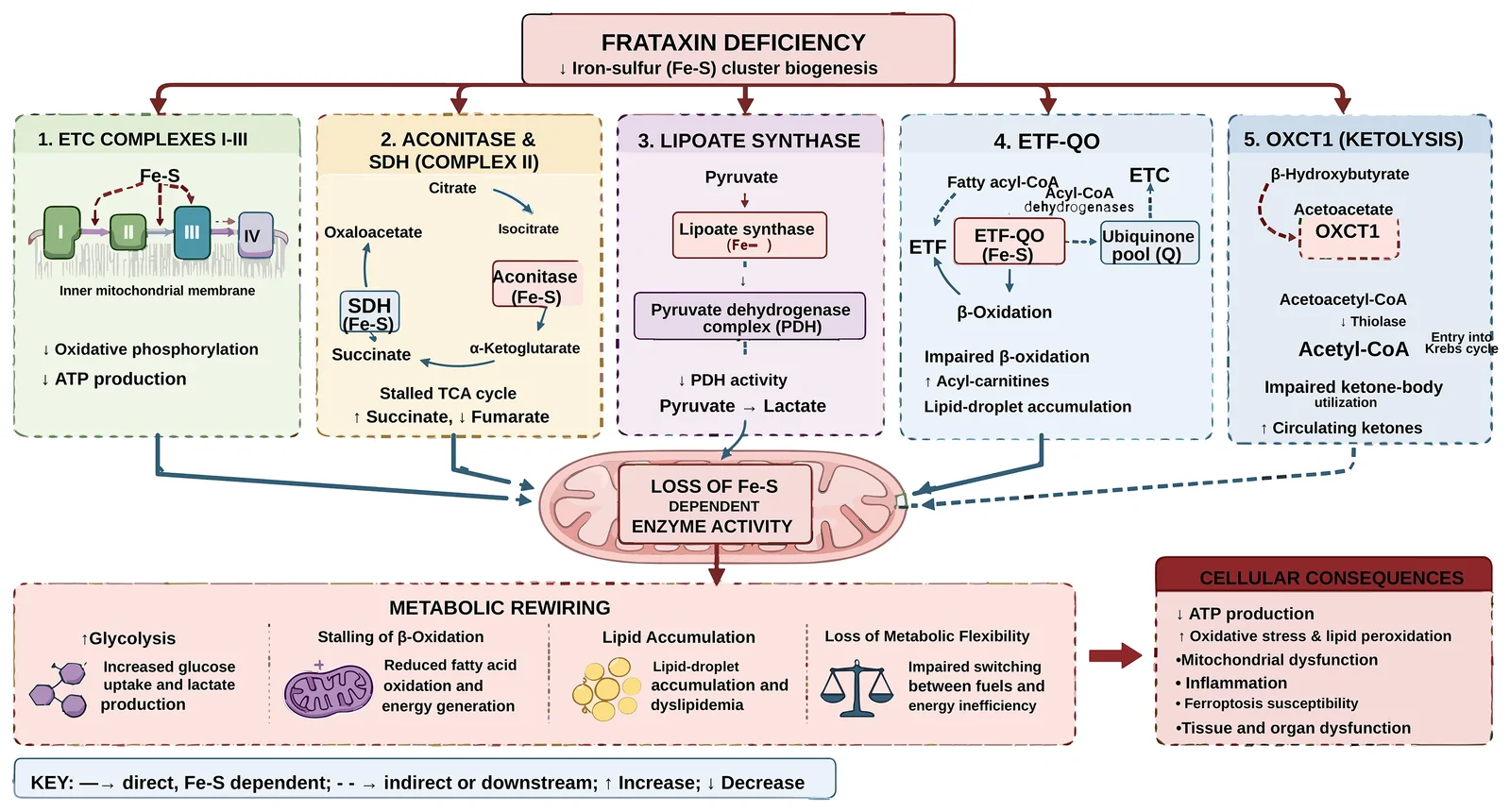
Iron–sulfur cluster loss and the rewiring of fuel metabolism. Fe–S, iron–sulfur; ETC, electron transport chain; SDH, succinate dehydrogenase; PDH, pyruvate dehydrogenase; ETF-QO, electron-transfer-flavoprotein-ubiquinone oxidoreductase; OXCT1, 3-oxoacid CoA-transferase 1. Solid arrows indicate direct, Fe–S-dependent steps; dashed arrows indicate indirect or downstream relationships. The ETF-QO and ketolysis arrows have been completed to show electron flux from beta-oxidation into the ubiquinone pool and carbon flux from ketone bodies through OXCT1 into the Krebs cycle.

The secondary deficiency of OXCT1 deserves particular attention because it has recently been resolved mechanistically. Frataxin physically binds OXCT1 and stabilizes it by blocking its ubiquitination, an interaction that maps to the N-terminal 1–40 residues of frataxin; loss of frataxin therefore accelerates the proteasomal turnover of OXCT1 and impairs ketolysis, elevating circulating ketone bodies [58]. Paradoxically, the resulting ketosis may be partly adaptive: in the KIKO mouse, lowering OXCT1 to drive a chronic mild ketosis raised frataxin levels and improved neurobehavioral performance, apparently through β-hydroxybutyrate-driven enhancement of mitochondrial biogenesis [58]. This duality, ketone-utilization failure as a deficit but ketone exposure as a beneficial signal, frames ketone metabolism as both a vulnerability and a potential lever for therapy in FRDA.

Energy failure in FRDA is compounded by disordered mitochondrial calcium handling. Matrix calcium normally stimulates Krebs-cycle dehydrogenases and respiratory complexes to match ATP output to demand; in frataxin-deficient lymphoblasts, cardiomyocytes and dorsal root ganglion neurons, compensatory downregulation of the mitochondrial sodium-calcium exchanger NCLX raises matrix calcium in an attempt to sustain ATP production, but ultimately pushes calcium past the threshold that opens the mitochondrial permeability transition pore and triggers cell death [56]. Reduced expression of the uniporter subunit (mitochondrial calcium uniporter) MCU in patient skeletal muscle [56] suggests that calcium uptake is also constrained, leaving mitochondria caught between insufficient calcium-stimulated respiration and calcium-overload-triggered death. These calcium derangements provide a mechanistic bridge between the bioenergetic deficit and the selective vulnerability of high-demand neurons and cardiomyocytes.

### 3.2. Lipid metabolism

Many studies have implicated abnormal lipid metabolism in FRDA, though the exact metabolic enzymes altered have not been fully defined. Such investigations include not only early pregenetic era studies, but also more recent investigations of lipid levels and plasma lipoprotein levels [57,59]. The diffuse nature of such dysfunction in FRDA has come from transcriptomic, proteomic and metabolic studies across many tissues, including fibroblasts, peripheral blood mononuclear cells (PBMC) ventricular myocytes and iPSC-derived cells [59–64]. A variety of miscellaneous lipid abnormalities have appeared associated with deficient frataxin levels, such as a selective deficit of cardiolipin and deficiency of steroid biosynthesis [65,66].

Among the most reproducible lipid abnormalities is the accumulation of neutral-lipid droplets, observed in frataxin-deficient *Drosophila* glia, in the livers of conditional knock-out mice (where steatosis is accompanied by giant, cristae-poor mitochondria), and in striated-muscle and cardiac frataxin-deficient models [59–64,67]. In frataxin-deficient cardiomyocytes, droplet accumulation coincides with the induction of medium-chain acyl-CoA dehydrogenase and carnitine palmitoyl transferase I, indicating that lipid is being mobilized for oxidation faster than it can be consumed, a futile catabolic cycle that leaves lipid stranded in droplets while ATP remains scarce [64]. This pattern, conserved from flies to the mammalian heart, supports the view that the primary lesion is a failure to complete β-oxidation rather than a failure to engage it.

In addition, selected increases in ceramides and sphingolipids appear [57,68,69]. These are potentially directly toxic to cells and may reflect a relative absence of the catabolism of medium- to long-chain fatty acids as well as selected increases in the enzymes needed for ceramide synthesis. Similarly, the palmitoylation of selected proteins (transferrin receptor and synaptic proteins) is altered in peripheral cells and neurons; this may explain some of the loss of soluble transferrin receptor in FRDA, although this may also be linked to other phenomena [70–72]. More broadly, this ceramide increase reflects disrupted fatty acid handling, in which impaired β-oxidation leaves fatty acids and their acyl-CoA forms in excess, evident as elevated acylcarnitines, HMG-CoA, and β-hydroxybutyryl-CoA and as accumulating lipid droplets, and this surplus substrate, together with increased de novo synthesis, drives ceramide accumulation, consistent with the ceramide elevation reported in frataxin-deficient heart and in patient fibroblasts (figure 3).

**Figure 3.**
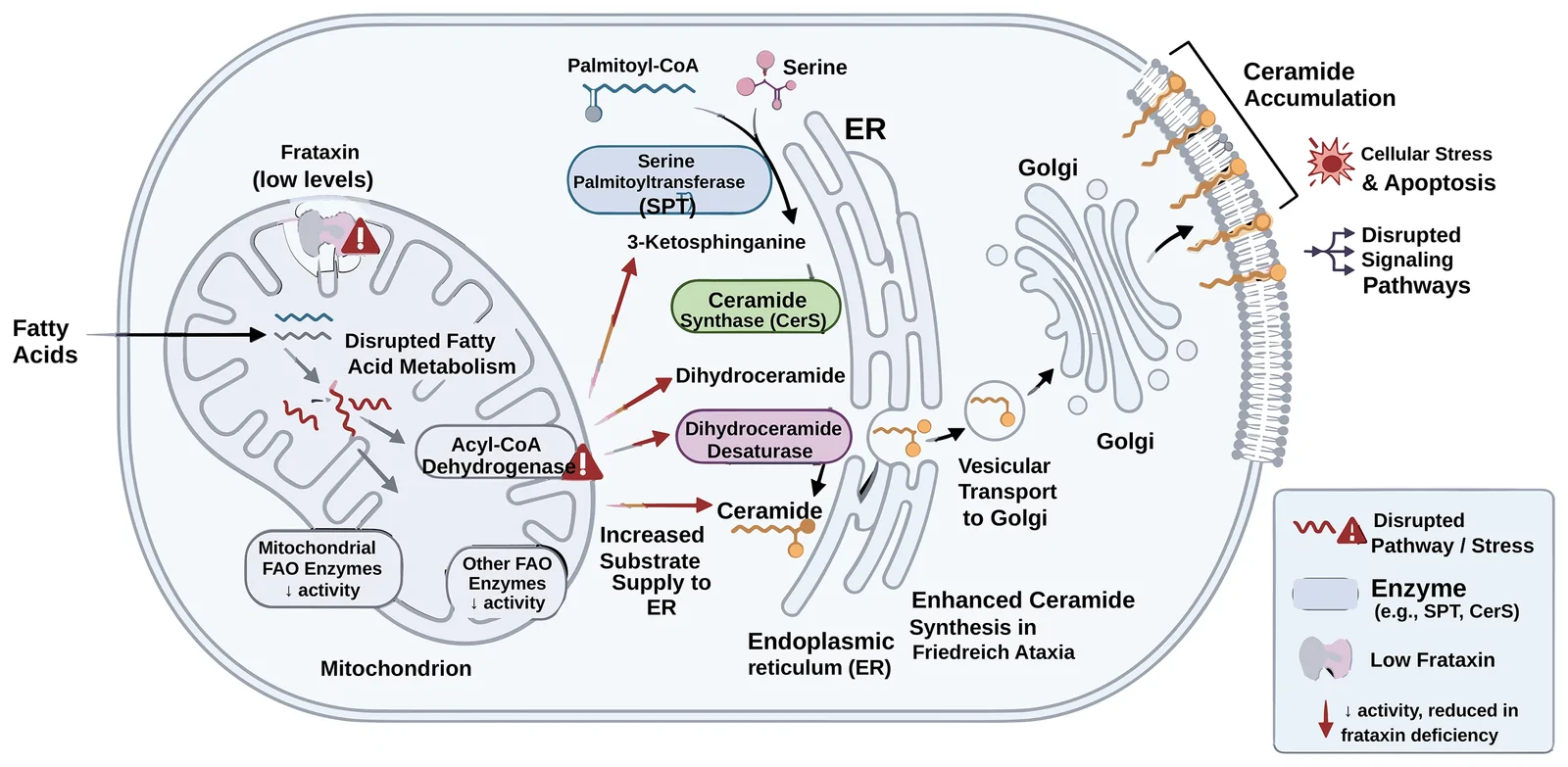
Disrupted fatty acid handling feeds ceramide synthesis in Friedreich ataxia. Fe–S, iron–sulfur; SPT, serine palmitoyltransferase; CerS, ceramide synthase; HMG-CoA, 3-hydroxy-3-methylglutaryl-CoA; BHB-CoA, β-hydroxybutyryl-CoA. Enzymes are drawn as rounded boxes and shaded by their status in frataxin deficiency, with reduced activity or abundance in blue and increased activity or abundance in pink; the enzyme symbol has been added to the figure key. Mitochondrial beta-oxidation enzymes and ETF-QO are marked as reduced, whereas MCAD, CPT1, SPT and the ceramide synthases are marked as induced.

The sphingolipid disturbance in FRDA is more specific than a generic increase in ceramides. In patient fibroblasts, several ceramide species rise in proportion to GAA-repeat length and inversely with frataxin level [57], whereas in plasma, the dominant change is a selective depletion of very-long-chain (VLC) ceramides, with long-chain species relatively preserved, so that the long-chain-to-VLC ceramide ratio rises and discriminates patients from controls [73]. Modelling in iPSC-derived cardiomyocytes traced this signature to preferential impairment of ceramide synthase 2 (CerS2), the enzyme that elongates ceramides into VLC species, with frataxin knockdown enriching long-chain and depleting VLC ceramides [73]. Because this ratio shift arises mainly from VLC depletion rather than long-chain elevation, it is mechanistically distinct from the ceramide accumulation described in common metabolic diseases and points towards an elongation or supply defect, raising the prospect of a circulating, chain-resolved ceramide biomarker for FRDA.

### 3.3. Other cellular metabolic abnormalities in FRDA

Beyond lipids, untargeted metabolomics has revealed coordinated disturbances in central-carbon and one-carbon metabolism. Plasma from FRDA patients shows dysregulated one-carbon metabolism, with strikingly narrow variances in metabolites such as formate and sarcosine that suggest a tightly constrained, disease-specific pathway rather than incidental variation [74]. Integrated multi-omic profiling of plasma, combining metabolomics, lipidomics and proteomics, further implicates dysregulated sphingolipid, phospholipid, citric acid cycle, amino acid and apolipoprotein metabolism, and has been used to build classifiers that distinguish patients from controls and carriers [73]. Such systems-level signatures reinforce the breadth of metabolic involvement in FRDA and offer a route to circulating biomarkers of disease state and treatment response.

FRDA and downstream metabolic modulators: frataxin deficiency and its compensatory mechanisms alter a variety of downstream signalling pathways, including sirtuins, PGC-1alpha, glutathione signalling and NRF2. Sirtuins are a family of NAD⁺-dependent protein deacylases and ADP-ribosyltransferases that regulate diverse cellular processes, including metabolic homeostasis, inflammatory responses, oxidative stress and apoptosis. Among the seven identified mammalian sirtuins, SIRT1, SIRT6 and SIRT7 are predominantly localized in the nucleus, SIRT2 in the cytoplasm, and SIRT3, SIRT4 and SIRT5 in the mitochondria. SIRT1, SIRT2 and SIRT3 are robust protein deacetylases and are generally considered the major deacetylating sirtuins. SIRT6 possesses deacetylase activity but is often more efficient at removing long-chain fatty acyl groups and also regulates chromatin through histone deacetylation. SIRT4, SIRT5 and SIRT7 exhibit weak or no detectable deacetylase activity. In frataxin-deficient DRG neurons, the loss of frataxin disrupts electron transport chain function, reducing the mitochondrial NAD⁺/NADH ratio and impairing SIRT3 activity. Consequently, its downstream target superoxide dismutase (SOD2), a potent mitochondrial antioxidant enzyme, becomes hyperacetylated and inactivated, promoting oxidative stress [75]. Reduced SIRT3 activity also causes hyperacetylation of cardiac mitochondrial proteins, which is temporally progressive and parallels the evolution of heart failure in the *FXN* KO model [76,77]. Among sirtuins, SIRT3 levels decrease in heart and dorsal root ganglia in some models [75–77], and the N46S polymorphism in SIRT6, an enzyme controlling the switch between carbohydrate and fatty acid metabolism, is associated with differential speed of progression of neuronal disease in FRDA [78]. Similarly, PGC-1alpha signalling is deficient in neurons in FRDA, providing a mechanism for decreases in lipolysis in FRDA as well as selective effects on Purkinje cells [51,79–84].

The route by which these pathways fail in FRDA matters for therapy, because neither the sirtuins nor NRF2 is mutated in the disease. Sirtuin activity is set by the availability of NAD+, so any lesion that lowers the mitochondrial NAD+ to NADH ratio reduces deacetylase flux without changing sirtuin protein levels. Two such lesions operate together in frataxin deficiency. Loss of the Fe–S-dependent complexes I–III leaves NADH unoxidized and depletes free NAD+, and the parallel loss of PGC-1alpha signalling reduces transcription of the mitochondrial biogenesis program that SIRT3 serves [79–84]. Substrate limitation and reduced enzyme expression therefore compound one another, and the result is progressive hyperacetylation of SIRT3 targets, including SOD2, the fatty acid oxidation and Krebs-cycle enzymes [75–77]. Because hyperacetylation further impairs both antioxidant defence and fuel oxidation, the loop is feed-forward rather than static. This mechanism predicts that NAD+ repletion should restore deacetylase flux without correcting frataxin, providing a rationale for the NAD+ precursor and exercise trials discussed below [85,86].

Consistent with the ROS susceptibility of cells in FRDA, the cellular antioxidant glutathione appears depleted along with other antioxidant defences under the control of the transcription factor NRF2. NRF2 is a master regulator of antioxidant gene expression that binds to antioxidant response elements (AREs) in the promoters of target genes, including those encoding glutathione-related enzymes and superoxide dismutase. Under basal conditions, NRF2 is retained in the cytosol through its interaction with Kelch-like ECH-associated protein 1 (*KEAP1*), which is tethered to actin bundles [87]. Oxidative stress causes NRF2 to detach from *KEAP1* and translocate into the nucleus, where it activates the expression of antioxidant genes [88]. In FRDA, however, NRF2 signalling is impaired. Both NRF2 mRNA and protein levels are reduced, accompanied by disorganization of the actin cytoskeleton and increased actin glutathionylation, which further hinders NRF2 nuclear translocation [80,88]. While ROS production in cells normally activates NRF2 signalling, it is paradoxically suppressed in FRDA cells, leading to decreased levels of NRF2 and inability to enter the nucleus [89–91]. As a result, ARE element-containing genes are not activated in response to ROS, leading to depletion of glutathione and other defences. The exact reason for this failure to activate NRF2 is unclear, though it appears in multiple different tissues. Several non-exclusive mechanisms have been proposed, and they are experimentally separable. *KEAP1* is tethered to the actin cytoskeleton, and in FRDA the cytoskeleton is disorganized and actin is glutathionylated, so NRF2 remains sequestered even when oxidants accumulate [87,92]. Signalling into the pathway is also altered, since GSK3beta drives nuclear exclusion and degradation of NRF2 through a *KEAP1*-independent route, while impaired LKB1 and AMPK signalling removes a stimulus that normally favours nuclear retention [90]. Transcription of *NFE2L2* itself is reduced, so the pool available for translocation is smaller before any of these steps apply [89,90]. Because Omaveloxolone acts at the *KEAP1* cysteine sensor, the relative weight of these mechanisms determines how much of the pathway can be recovered pharmacologically, which makes resolving them a priority for non-frataxin-directed therapy.

The suppression of NRF2 has a direct corollary in ferroptosis, an iron-dependent form of regulated cell death driven by unchecked lipid peroxidation. In frataxin-deficient dorsal root ganglion neurons and in the FXNI151F mouse, transferrin-receptor-1 upregulation and ferritin loss drive mitochondrial iron accumulation, while impaired nuclear translocation of NRF2 downregulates its targets *SLC7A11* (the cystine transporter that supplies glutathione synthesis) and glutathione peroxidase 4 (GPX4), the principal defence against lipid peroxides; the combination lowers the ferroptotic threshold, with LKB1/AMPK (adenosine monophosphate), *KEAP1* and GSK3β implicated in the failed NRF2 response [93]. Ferroptosis thus links the iron, redox and lipid arms of FRDA pathophysiology into a single death pathway, and it rationalizes both NRF2-directed therapy and emerging interest in direct ferroptosis inhibitors and lipid-peroxidation-resistant fatty acids. Mitochondrial iron accumulation, driven by transferrin-receptor-1 upregulation and ferritin loss, together with impaired NRF2 signalling that lowers the cystine transporter system xc- and glutathione peroxidase 4, depletes glutathione and allows lipid peroxidation to proceed to ferroptotic cell death, with collapse of the GSH : GSSG and NAD+ : NADH ratios indexing this redox state in blood (figure 4).

**Figure 4.**
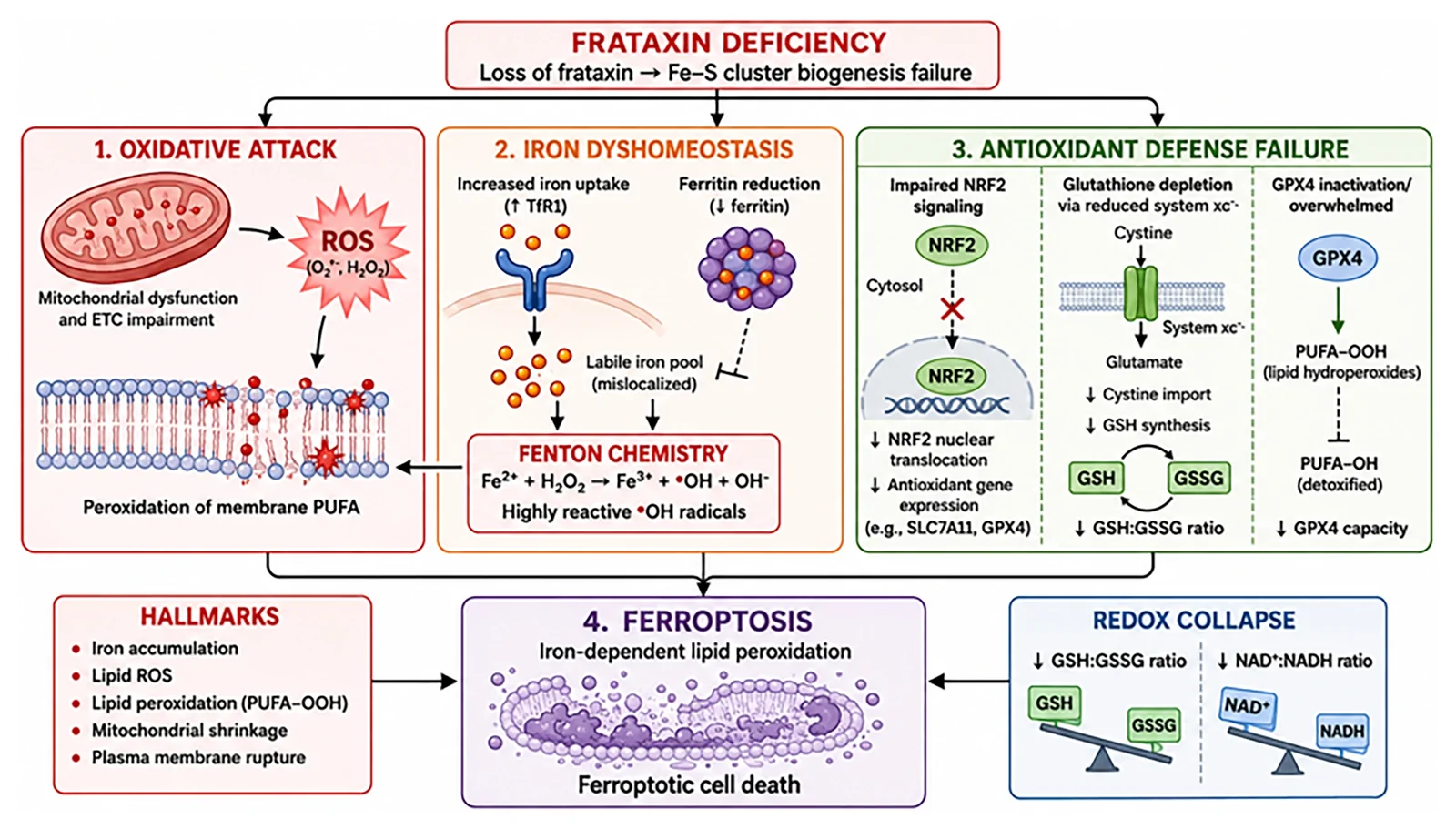
Redox collapse and ferroptosis in Friedreich ataxia. NRF2, nuclear factor erythroid 2-related factor 2; GSH, reduced glutathione; GSSG, oxidized glutathione; GPX4, glutathione peroxidase 4; PUFA, polyunsaturated fatty acid; ROS, reactive oxygen species.

## 4. Metabolism in individual tissues in Friedreich ataxia

### 4.1. Cardiac metabolism

Because cardiomyopathy is the leading cause of death in FRDA, the metabolism of the frataxin-deficient heart merits separate consideration [94]. Approximately 60% of patients develop a hypertrophic cardiomyopathy that, unlike the sarcomeric hypertrophic cardiomyopathies, reflects massive proliferation of abnormal mitochondria within the cardiomyocyte rather than overexpression of contractile proteins [95]. The hypertrophy is typically concentric and non-obstructive, and over time the heart develops chronic cardiomyocyte apoptosis, interstitial and replacement fibrosis, and diastolic dysfunction; in later stages, the ventricle may dilate with a falling ejection fraction and overt heart failure. Cardiac causes account for roughly 80% of deaths in FRDA [94], yet the heart has been studied far less systematically than the nervous system, and its early natural history, especially in children, remains poorly defined.

A consistent theme is that the energetic deficit precedes overt structural disease. Phosphorus-31 magnetic resonance spectroscopy shows that the cardiac phosphocreatine-to-ATP ratio is reduced by approximately 40% in FRDA hearts, even in the absence of hypertrophy or dysfunction, and myocardial perfusion reserve is impaired even in the youngest patients and in hearts without hypertrophy, a pattern that resembles the heart in diabetes or metabolic syndrome [95]. In this sense, the failing FRDA heart is a paradigm of an engine out of fuel: when energy cannot be produced efficiently through fatty acid oxidation, the Krebs cycle and the electron transport chain, the myocardium must rely on less efficient pathways such as glycolysis. It also loses the metabolic flexibility that normally allows it to switch among substrates. In the frataxin-deficient cardiomyocyte, this energetic failure is accompanied by the proliferation of abnormal mitochondria rather than sarcomeric overgrowth, SIRT3-dependent hyperacetylation of fatty acid oxidation and Krebs-cycle enzymes, a positron-emission-tomography-documented switch from fatty acid to glucose utilization, chronic troponin leak and a coronary microvasculopathy that limits perfusion reserve. In combination, these changes produce the hypertrophic cardiomyopathy that is the leading cause of death in the disease (figure 5).

**Figure 5.**
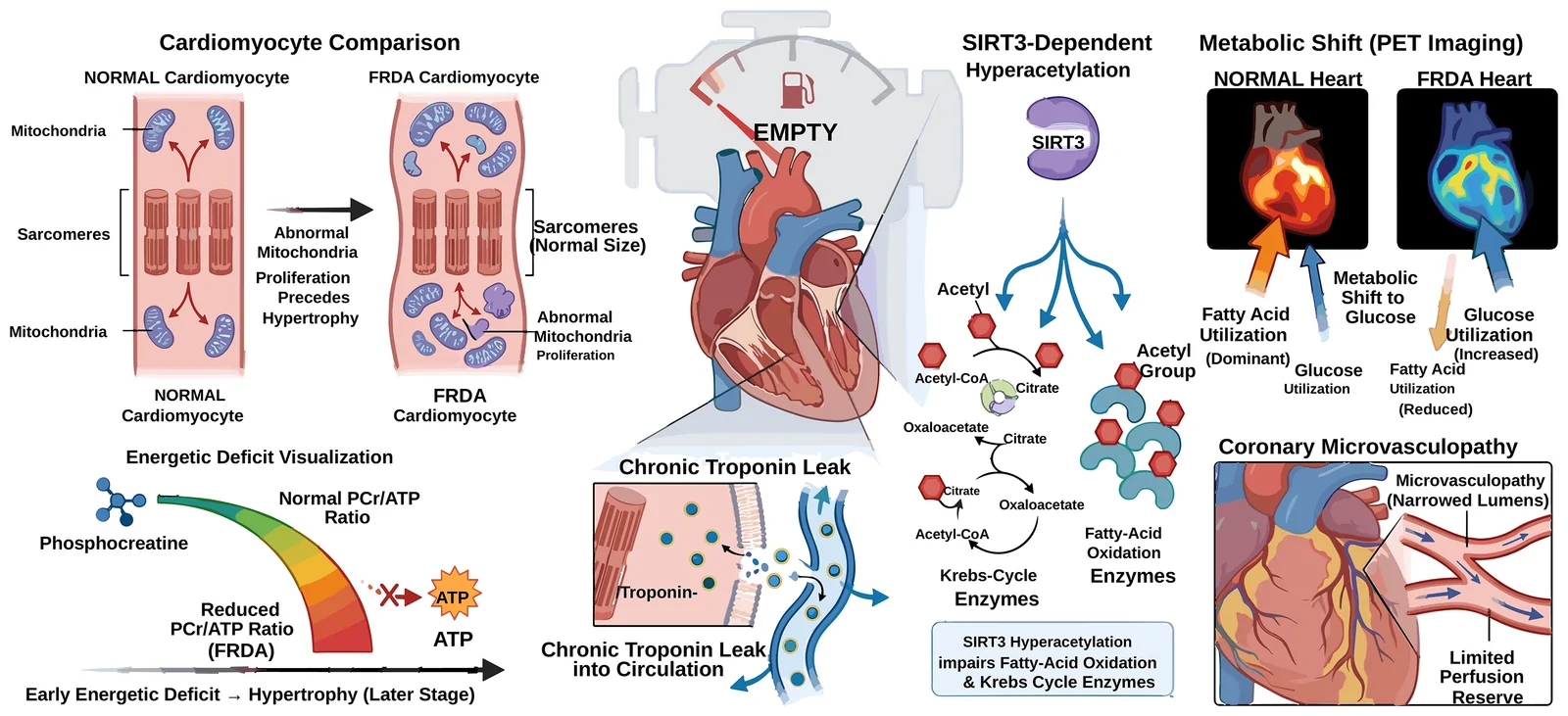
The frataxin-deficient heart as an engine out of fuel. PCr, phosphocreatine; PET, positron emission tomography; FAO, fatty acid oxidation; SIRT3, sirtuin 3.

A central molecular contributor is the loss of SIRT3 activity. Frataxin-deficient hearts accumulate acetylated mitochondrial proteins, including the fatty acid oxidation and Krebs-cycle enzymes whose activity SIRT3 normally restores by deacetylation, and the degree of hyperacetylation tracks with the progressive loss of cardiac function [76,77]. At the structural level, frataxin-deficient cardiomyocytes accumulate lipid droplets and induce medium-chain acyl-CoA dehydrogenase and carnitine palmitoyl transferase I, the same stranded-lipid pattern seen in other tissues and consistent with fatty acids being mobilized for oxidation faster than they can be consumed.

Direct evidence that the human FRDA heart shifts its fuel preference now comes from positron emission tomography. In a fasting study using fluorodeoxyglucose and 11C-palmitate, most FRDA participants showed increased myocardial glucose uptake relative to controls, and patients separated into two metabolically distinct subclasses by left-ventricular mass; those with greater hypertrophy showed the highest glucose-to-palmitate utilization together with troponin leak and impaired systolic and diastolic function [96]. Transcriptomics of the frataxin-deficient mouse heart corroborated the shift, with upregulation of glucose-uptake and glycolytic genes and downregulation of genes for mitochondrial energy production. These findings convert the long-hypothesized fatty-acid-to-glucose switch into a measurable phenotype and suggest that myocardial substrate imaging could stage cardiac involvement and report on metabolically targeted therapy.

Several additional features link cardiac metabolism to outcome. Many patients chronically leak cardiac troponin in the absence of symptoms, which likely reflects ongoing cardiomyocyte injury and may serve as a biomarker of disease activity, particularly in the hypertrophic heart [91]. The abnormal, proliferating mitochondria are not efficiently cleared, raising the possibility that defective mitophagy contributes to their accumulation, and the small intramural coronary arteries are narrowed, producing a microvasculopathy that limits the perfusion reserve needed to meet metabolic demand. Consistent with a SIRT3-centred lesion, restoring NAD+ improves cardiac bioenergetics and function in FRDA models, and NAD+-precursor supplementation has advanced into clinical testing [85,86]. Finally, the FRDA heart is not purely cell-autonomous, as cardiac fibroblasts and other non-myocyte populations appear to convey components of the pathophysiology to cardiomyocytes [28,97].

### 4.2. Neural metabolism

The nervous system shows the same loss of metabolic flexibility as the heart, but in a different set of vulnerable cells. The earliest pregenetic studies already pointed to a fuel problem, since glycolytic enzyme activity was reduced in the nucleus dorsalis of Clarke, the deep cerebellar nuclei, and Purkinje cells, and pyruvate oxidation was impaired [29–32]. Modern imaging supports the same conclusion, with low complex I signal and altered neurochemical profiles in the cerebellum of patients and of mouse models [49,50,98]. The two neuronal populations that define the clinical phenotype, the large proprioceptive neurons of the dorsal root ganglia and the large glutamatergic neurons of the dentate nucleus, are also those with the highest oxidative demand and the least capacity to compensate through glycolysis. Ketone bodies are a particular problem for these cells, because the secondary loss of OXCT1 blocks ketolysis and proprioceptive neurons require ketolysis for normal development [54,55]. Frataxin-deficient dorsal root ganglion neurons additionally show reduced SIRT3 activity, hyperacetylation of SOD2, mitochondrial iron accumulation, impaired NRF2 translocation and a lowered ferroptotic threshold, so the redox and lipid arms of the disease converge on the neurons that fail clinically [75,93].

Neural metabolism is not uniform across cell classes, and frataxin abundance predicts vulnerability only in part. Neurons carry higher frataxin levels than glia, and among neurons the large projection cells are the richest, including cerebellar Purkinje cells, the large glutamatergic neurons of the dentate nucleus and the large proprioceptive neurons of the dorsal root ganglia [98–100]. Astrocytes, microglia and oligodendrocytes express frataxin at lower levels, but they are not passive in the disease. Frataxin knock-down in human astrocytes produces mitochondrial fragmentation, superoxide generation, p53-dependent cell death and secretion of factors that are toxic to co-cultured neurons, which places glia upstream of part of the neuronal lesion [101]. Microglial and broader immune contributions are considered in the next section. Because astrocytes supply neurons with lactate and support their lipid handling, the metabolic lesion in FRDA brain is best read as a failure of the neuron-glia metabolic unit rather than of neurons alone, and this reading also predicts that a therapy acting only on neurons will address only part of the problem.

### 4.3. Organ level and interorgan metabolic events in Friedreich ataxia

While frataxin is found in all cells, its levels vary throughout the body, with high levels in the brain, heart, liver, dorsal root ganglia, pancreatic islet cells and muscle. Lower levels are found in blood cells, epithelia and replicating tissue such as fibroblasts [98,99]. In the brain, levels are high in the cerebellum in general, particularly in Purkinje cells [100]. Thus, the tissue distribution corresponds to the locations of cell death in FRDA suggesting that FRDA is largely (but not necessarily exclusively) a cell- and organ-autonomous disease [98–100]. Consistent with this, cardiac transplantation does not ameliorate neurologic disease, and the severity of involvement of individual organs is linked to GAA repeat length, but after accounting for GAA1 repeat length, each organ system changes independently [102,103]. Similarly, in mice, organ-specific reductions are sufficient for reproducing the pathology of the disease, suggesting that non-cell autonomous events are not required [97,104,105]. However, there are exceptions to these ideas. In the cerebellum, Purkinje cells make the highest levels of frataxin but are less affected than dentate nucleus cells [98–100]. In addition, numerous systemic and localized immune phenomena may augment the pathophysiology of the disease. This includes evidence from transcriptomic approaches examining peripheral PBMCs, markers of gliosis-related activation in the brain and linkage of these brain microglial changes to metabolic changes in microglia [106–110]. Consequently, even though FRDA appears as an organ autonomous disease for the most part, intercellular metabolic communication may represent a therapeutic target.

The best characterized interorgan metabolic phenomenon in FRDA is diabetes mellitus (DM), which has features of both insulin deficiency and decreased insulin sensitivity in FRDA [111–116]. Beta cells are subject to apoptosis with frataxin deficiency, leading to either their loss *in vivo* or with failure to develop. This process is not immune-mediated, and patients have no islet cell antibodies. In parallel, the mitochondrial dysfunction of FRDA leads to decreased muscle insulin sensitivity. This is particularly associated with a deficiency of PGC1a and AMP kinase [117], and in animal models, the insulin sensitivity is associated with failure of lipolysis before failure of glucose utilization [115]. In humans, lower oxidative phosphorylation activity levels, inactivity and visceral adiposity are associated with lower insulin sensitivity. As a result, glucose tolerance tests may be abnormal in the majority of FRDA patients, though frank diabetes is identified in only about 10% [83,84,117]. DM itself is more common in patients with long GAA repeat lengths and earlier age at presentation. In general, the later the onset of FRDA, the later the onset of diabetes, and the more features of decreased insulin sensitivity characterize DM in FRDA. Still, diabetes in FRDA can be associated with diabetic ketoacidosis (DKA) even in later onset. The treatment of DM in FRDA is typical of other DM patients: diet, weight control, oral agents and insulin, though almost all early-onset DM patients will require insulin. Individuals with DM in FRDA progress faster, though this may not reflect a specific phenomenon. Interestingly, heterozygous expression of an expanded GAA appears to serve as a risk factor for decreased insulin sensitivity, consistent with a larger role of frataxin in insulin sensitivity.

Other interorgan metabolic interactions are likely to exist in FRDA but have not yet been explored. For example, many FRDA patients have osteopenia or osteoporosis, even in the time before losing ambulation, suggesting dysfunctional metabolic control of bone density [24,25]. Other such interactions will become better understood over time.

Another underappreciated component of intercellular metabolic dysfunction in FRDA is the role of the liver. The liver is viewed as unaffected in FRDA, perhaps reflecting the ability of the liver to regenerate. However, hepatic iron stores are decreased in FRDA in both humans and animal models [118,119]. Metabolic studies using 13C breath tests also show diminished function, and early studies suggested decreased hepatic lipase activity [120]. A variety of hepatic proteins are decreased in FRDA, including lipoproteins and ferritin, suggesting a subclinical role of the liver in FRDA, or alternatively, the existence of a compensatory or regulatory pathway involving the liver. The organs involved in FRDA are those that use fatty acids, or their breakdown products, the ketone bodies, as cellular fuels, all of which are supplied by the liver. Consequently, the effect of frataxin deficiency on the liver and its protein products may not always be subclinical, particularly for symptoms like fatigue that may depend on the ability of tissues to produce energy from liver-supplied substrates.

The hepatic contribution may be best understood as a supply side problem. The tissues that fail in FRDA, namely the heart, skeletal muscle and the nervous system, are precisely those that import fatty acids and their ketone-body derivatives as fuel, and the liver is the organ that packages and exports those substrates. Frataxin-deficient liver and frataxin-knock-down hepatocyte models show reduced apolipoprotein A-I, the principal protein of HDL, mirroring the low circulating ApoA-I in patients [59], alongside diminished lipoprotein lipase activity and reduced hepatic mitochondrial function on 13C breath testing. If a frataxin-deficient liver exports altered lipoprotein and ceramide cargo to the periphery, a second, supply-side hit could compound the cell-autonomous cardiac lesion, reconciling the tissue ceramide elevations with the depletion of VLC ceramides in the blood as a single shift in lipid export and handling read across compartments. This hypothesis remains to be tested directly, but it reframes the liver from a bystander into a candidate driver of the systemic and fatigue-related features of the disease.

Adipose tissue provides a parallel example of frataxin shaping whole-body fuel supply. In a frataxin-deficient mouse model, lipolysis rather than glucose handling is the earliest disrupted pathway in white adipose tissue, with reduced adipose triglyceride lipase and hormone-sensitive lipase, adipocyte enlargement and inflammation preceding hyperglycemia by months; pharmacologic activation of lipolysis or of PPARγ restored insulin sensitivity [83]. In adults with FRDA, reduced skeletal-muscle insulin sensitivity has likewise been documented and linked to oxidative-phosphorylation capacity [121]. Together, these data place insulin resistance downstream of a primary defect in lipid mobilization and oxidation, consistent with the broader theme that FRDA is a disease of fuel logistics as much as of fuel combustion.

## 5. Clinical aspects of metabolism in Friedreich ataxia

### 5.1. Metabolic biomarkers in FRDA

The metabolic map of FRDA also defines a panel of blood-based biomarkers, an acute unmet need given that physiological improvement during treatment can occur without measurable change in frataxin or in standard clinical markers. A natural design anchors such a panel on the frataxin protein itself, now quantifiable by isotope-dilution mass spectrometry in whole blood [122], against which downstream metabolic readouts are benchmarked in the same sample. The ceramide signature supplies the lipid axis: VLC ceramides are depleted, and the long-chain-to-VLC ratio rises in FRDA whole blood, discriminating patients from controls with areas under the curve of roughly 0.75–0.85 for individual species and above 0.9 for a multi-metabolite model, while correlating directly with frataxin in the same samples [73].

Three further axes extend the panel beyond lipids and map onto the cellular lesions described above. Because succinate dehydrogenase (complex II) is an Fe–S enzyme and an early casualty of frataxin loss, succinate accumulates while fumarate falls, so the succinate : fumarate ratio offers a plasma-accessible surrogate of complex II activity; the same ratio separates *SDHx* variant carriers from controls with areas under the curve of 0.86–0.95 [123], a mechanistically homologous lesion. The secondary loss of OXCT1 impairs ketolysis and raises circulating ketone bodies, so the BHB : acetoacetate ratio reports on mitochondrial fuel flexibility [54]. The redox collapse of FRDA is captured by the NAD+ : NADH and GSH : GSSG ratios; NAD+ metabolites are pharmacodynamically responsive to precursor supplementation and exercise [96], and reduced glutathione is measurably lower in frataxin-deficient cells and tissues [87,88].

The value of these measures lies less in any single analyte than in their integration. Anchored to frataxin and combining the ceramide ratio with the succinate : fumarate, BHB : acetoacetate, NAD+ : NADH and GSH : GSSG ratios, a compact panel drawn from one whole-blood sample could capture the breadth of FRDA metabolic dysfunction, improve discrimination over any single marker, and provide pharmacodynamic sensitivity across therapeutic mechanisms, including those not expected to raise frataxin. Table 1 summarizes the principal metabolic nodes, their candidate blood biomarkers and the therapeutic targets that emerge from each.

**Table 1.** Principal metabolic nodes of frataxin deficiency, their candidate blood-based biomarkers, and emerging therapeutic targets in Friedreich ataxia. Arrows denote the direction of change relative to unaffected controls. Sample sizes are given where the primary study reports a defined cohort; entries describing model systems denote preclinical work. LC, long-chain; VLC, very-long-chain.

metabolic node	key enzyme/metabolite	change	model/cohort (*n*)	candidate blood biomarker	candidate therapeutic target	reference(s)
oxidative phosphorylation	ETC complexes I–III (Fe–S subunits)	ATP ↓, lactate ↑	human cohort studies, cardiac and skeletal muscle 31P-MRS and CrCEST MRI	PCr : ATP ratio (imaging)	NAD+ repletion; ETC support	[16–19,93]
TCA cycle/complex II	succinate dehydrogenase, aconitase	succinate ↑, fumarate ↓	Fe–S enzyme assays in patient cells and tissue; SDHx plasma cohort	succinate : fumarate ratio	anaplerotic support	[43–50,52,124]
fatty acid β-oxidation	ETF-QO; MCAD/CPT1; lipid droplets	β-oxidation stalled; acyl-carnitines ↑	nine FRDA and nine control fibroblast lines; rat and mouse cardiomyocytes; *Drosophila*	acyl-carnitines	lipid-handling/PPAR agonism	[57,59–64,67]
ketone-body oxidation	OXCT1	fetolysis impaired; BHB ↑	*n* = 5–6 per cell experiment; *n* = 4–7 mice per group	BHB : acetoacetate ratio	ketone-ester/ketogenic therapy	[54,55,58]
sphingolipid remodelling	ceramide synthase 2 (CerS2)	VLC ceramides ↓; LC : VLC ratio ↑	nine FRDA and nine control fibroblast lines; 33 FRDA and 15 controls (plasma); 28 FRDA and 29 controls (whole blood)	LC : VLC ceramide ratio	ceramide-directed strategies	[57,68,69,73]
redox/NAD+	NAD+/NADH	NAD+ ↓; redox imbalance	randomized 2 × 2 factorial trial in FRDA patients; mouse cardiomyopathy model	NAD+ : NADH ratio	NAD+ precursors; exercise	[95,96]
glutathione/ ferroptosis	NRF2, SLC7A11, GPX4	GSH ↓; lipid peroxidation ↑	DRG neurons and FXNI151F mice; patient fibroblasts and blood	GSH : GSSG ratio	NRF2 activators; ferroptosis inhibitors; PPARγ	[87–91]
interorgan lipid supply	apolipoprotein A-I; lipoproteins	ApoA-I ↓	FRDA patient plasma; FXN-knockdown HepG2 hepatocytes	plasma ApoA-I	supply side (investigational)	[60,83,118–120]

### 5.2. Metabolism-based therapies for Friedreich ataxia

While correction of frataxin deficiency provides the most powerful therapies for FRDA, all of them at present have issues of distribution or safety; none is yet approved. Consequently, therapies based on metabolic changes in FRDA still provide approaches for amelioration of the features of FRDA. Omaveloxolone, a therapy based on reactivation of NRF2, is the only agent approved for FRDA, albeit presently only for individuals 16 and older. Omaveloxolone improves scores on a quantified neurological exam in a double-blind trial, with benefit extending for at least 3 years based on comparisons to natural history data [125,126]. Other agents like leriglitazone act on PPARgamma to increase mitochondrial biogenesis; it has shown mild benefit in a clinical trial [127]. Strategies based on NAD-based reversal of sirtuin abnormalities also show promise [95,96].

The pipeline beyond Omaveloxolone is now broad, and much of it is metabolically directed. Vatiquinone inhibits 15-lipoxygenase, the enzyme that initiates the lipid peroxidation cascade of ferroptosis, and it therefore targets the redox and lipid lesion described above. In the phase 3 MOVE-FA trial (NCT04577352), the change in total mFARS did not reach significance, but the upright stability subscale and the modified fatigue impact scale improved, and long-term extension data compared with matched natural history are consistent with slowing of progression. Leriglitazone acts on PPARgamma and improves iron homeostasis and ferroptotic markers in frataxin-deficient dorsal root ganglion neurons in addition to its effects on mitochondrial biogenesis [79,80,127]. Direct ferroptosis inhibitors, deuterated polyunsaturated fatty acids that resist peroxidation, and NAD+ precursors combined with exercise extend the same logic, and the last of these has now been tested in a randomized factorial trial [95,96]. Ketone esters and ketogenic strategies follow directly from the OXCT1 lesion, although the duality of impaired ketolysis alongside beneficial ketone signalling means that dose and timing will matter.

Frataxin-directed approaches are advancing in parallel, and they matter here because they set the comparator against which metabolic therapies will be judged. Protein replacement with nomlabofusp, a cell-penetrating peptide fused to frataxin, delivers frataxin to mitochondria and is in open-label extension testing. Gene therapy with LX2006, an adeno-associated viral vector carrying *FXN* and targeted to the heart, has raised cardiac frataxin and improved left-ventricular mass index and high-sensitivity troponin in early-phase studies (NCT05445323 and NCT05302271). Small molecules that override GAA-mediated silencing to raise *FXN* transcription are also in early clinical testing. None of these is yet approved, and each faces problems of tissue distribution, durability or immunogenicity. Metabolically directed therapy will therefore remain relevant, most plausibly as an adjunct that addresses the downstream lesions likely to persist once frataxin is only partially restored.

One further approach based on metabolism is anaplerosis, the use of alternative carbon sources to bypass metabolic blocks. Triheptanoin, a triglyceride of the seven-carbon fatty acid heptanoate, is approved for long-chain fatty acid oxidation disorders, and the breakdown of its odd-chain fatty acids fills the Krebs cycle at the level of propionyl-CoA [124,128,129]. Another anaplerotic approach could use amino acids, glutamine, for example, to enter the TCA cycle at other locations. Such an approach could theoretically lead to dietary therapy for FRDA, thus showing the ongoing relevance of understanding metabolism in this disease.

## 6. Conclusions and perspectives

The metabolic dysfunction of FRDA is coherent across scales. At the cellular level, the loss of Fe–S cluster biogenesis disables oxidative phosphorylation, the Krebs cycle, and fatty acid and ketone oxidation, forcing a glycolytic, lipid-stranded, ferroptosis-prone state. At the organ level, these defects manifest as a hypertrophic cardiomyopathy running out of fuel, a mixed diabetes, low bone density, and an underappreciated hepatic and adipose contribution to systemic fuel supply. Viewing FRDA simultaneously as a cell-autonomous Fe–S disease and as a disorder of interorgan fuel logistics clarifies why a globally reduced protein produces such a selective yet multisystem phenotype, and it identifies a set of tractable, non-frataxin-directed targets, including NRF2 activation, ferroptosis inhibition, NAD+ and sirtuin repletion, PPARγ agonism, anaplerosis and ketone-based therapy, which could ameliorate the disease while frataxin-restorative approaches mature.

## Data Availability

This article has no additional data.
